# Identification of Genes Expressed by Human Airway Eosinophils after an *In Vivo* Allergen Challenge

**DOI:** 10.1371/journal.pone.0067560

**Published:** 2013-07-02

**Authors:** Stephane Esnault, Elizabeth A. Kelly, Elizabeth A. Schwantes, Lin Ying Liu, Larissa P. DeLain, Jami A. Hauer, Yury A. Bochkov, Loren C. Denlinger, James S. Malter, Sameer K. Mathur, Nizar N. Jarjour

**Affiliations:** 1 Department of Medicine, Allergy, Pulmonary, and Critical Care Medicine Division, University of Wisconsin School of Medicine and Public Health, Madison, Wisconsin, United States of America; 2 Department of Pediatrics, University of Wisconsin School of Medicine and Public Health, Madison, Wisconsin, United States of America; 3 Department of Pathology, University of Texas Southwestern Medical Center, Dallas, Texas, United States of America; Murdoch University, Australia

## Abstract

**Background:**

The mechanism for the contribution of eosinophils (EOS) to asthma pathophysiology is not fully understood. Genome-wide expression analysis of airway EOS by microarrays has been limited by the ability to generate high quality RNA from sufficient numbers of airway EOS.

**Objective:**

To identify, by genome-wide expression analyses, a compendium of expressed genes characteristic of airway EOS following an *in vivo* allergen challenge.

**Methods:**

Atopic, mild asthmatic subjects were recruited for these studies. Induced sputum was obtained before and 48h after a whole lung allergen challenge (WLAC). Individuals also received a segmental bronchoprovocation with allergen (SBP-Ag) 1 month before and after administering a single dose of mepolizumab (anti-IL-5 monoclonal antibody) to reduce airway EOS. Bronchoalveolar lavage (BAL) was performed before and 48 h after SBP-Ag. Gene expression of sputum and BAL cells was analyzed by microarrays. The results were validated by qPCR in BAL cells and purified BAL EOS.

**Results:**

A total of 299 transcripts were up-regulated by more than 2-fold in total BAL cells following SBP-Ag. Mepolizumab treatment resulted in a reduction of airway EOS by 54.5% and decreased expression of 99 of the 299 transcripts. 3 of 6 post-WLAC sputum samples showed increased expression of EOS-specific genes, along with the expression of 361 other genes. Finally, the intersection of the 3 groups of transcripts (increased in BAL post SBP-Ag (299), decreased after mepolizumab (99), and increased in sputum after WLAC (365)) was composed of 57 genes characterizing airway EOS gene expression.

**Conclusion:**

We identified 57 genes that were highly expressed by BAL EOS compared to unseparated BAL cells after *in vivo* allergen challenge. 41 of these genes had not been previously described in EOS and are thus potential new candidates to elucidate EOS contribution to airway biology.

## Introduction

Recruitment of EOS to the lung has been reproducibly reported in allergic asthma [1]. While airway eosinophilia is commonly associated with increased risk for asthma exacerbation, severity, and poor prognosis [2]–[4], the precise correlation of EOS to the pathophysiology of asthma remains controversial. Reduction of airway EOS is associated with decline of submucosal matrix protein deposition and airway smooth muscle hyperplasia [5], [6] suggesting that EOS contribute to airway remodeling. Through the production and release of pro-inflammatory mediators, EOS can amplify the expression of Th1, Th2, and Th17 cytokines and chemokines [7]–[9] indicating they play a role in the adaptive immune response. Recent trials of anti-IL-5 antibodies (mepolizumab and reslizumab) have shown benefits in asthma, particularly in reducing rates of exacerbations [10]–[12].

One approach to understanding the biology of EOS in asthma is gene expression analysis by microarrays. Initial GeneChip analysis, which was performed using *in vitro* IL-5-activated circulating EOS, identified 66 genes that were up-regulated by IL-5 and predicted to have functions in adhesion, recruitment, activation and survival [13]. A subsequent study performed by our group showed that the expression of more than 200 genes was increased *in vitro* in IL-5- and GM-CSF-activated EOS, including the anti-apoptotic serine/threonine protein kinase Pim-1 [14], [15]. During their egress to the airway, in response to allergen, the phenotype of peripheral blood EOS changes dramatically [16]–[18]; however, gene analysis with microarrays of airway EOS has not been explored.

We performed gene expression array analysis on sputum samples obtained following whole lung allergen challenge (WLAC), and on bronchoalveolar lavage (BAL) cells obtained following segmental bronchoprovocation with an allergen (SBP-Ag). These two *in vivo* allergen challenge models are well-established asthma models that lead to eosinophilic airway inflammation [19]–[21]. Typically SBP-Ag causes EOS to increase in the BAL from ∼0.5% at baseline to ∼70% after allergen challenge [9] while WLAC induces an increase of EOS in sputum from ∼3% to ∼10% [21]–[24]. Therefore, we anticipated that analysis of total BAL and sputum cells by microarrays after SBP-Ag and WLAC and purified BAL EOS would facilitate identification of genes specifically expressed by airway EOS.

## Materials and Methods

### Subjects

The study was approved by the University of Wisconsin-Madison Health Sciences Institutional Review Board (IRB). Informed written consent was obtained from subjects prior to participation. Subjects had a history of mild atopic asthma as defined by at least one positive skin prick test and a history of allergen-induced asthma exacerbation with reversibility to albuterol >12% and/or a provocative concentration of methacholine producing a 20% fall in FEV_1_ (PC_20_) of <8 mg/ml. None of the subjects were using inhaled or oral corticosteroids. 10 subjects finished the SBP-Ag protocol and 2 were excluded for lower percentage of EOS in BAL after SBP-Ag (<67%) or low purity of BAL EOS (<99%). 18 subjects completed the sputum protocol and 12 were excluded due to having <500,000 cells, greater than 60% epithelial cells in the sputum, a greater than 2-fold difference in the percentage of epithelial cells pre- and post- WLAC, lack of RNA after extraction, or RNA integrity that was insufficient for microarrays.

### Bronchoscopy, SBP-Ag, and Anti-IL-5 Administration

**Figure 1:**
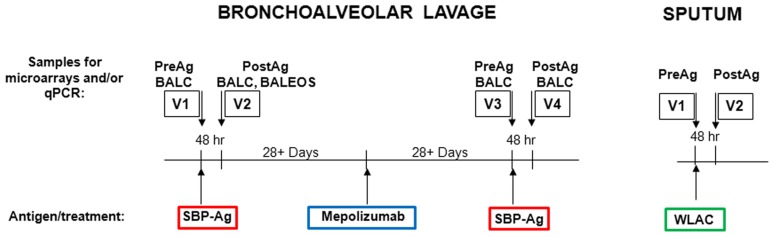
Bronchoalveolar lavage and sputum timelines. Atopic mild asthmatics underwent bronchoalveolar lavage (BAL) followed by segmental challenge with an allergen (SBP-Ag) on visit 1 (BAL V1). 48 h later on visit 2 (BAL V2), BAL was performed at the site of the challenged segment. One month later, a single dose of mepolizumab was administered. One month after dosing, BALs were repeated both before and after SBP-Ag. BAL cells (BALC) were prepared on visits 1, 2, 3, and 4 and airway EOS (BALEOS) were purified on visit 2. For the induced sputum study, sputum collection was performed followed by a whole lung allergen challenge (WLAC) on visit 1 (sputum V1). Collection of a second induced sputum was done 48 h after WLAC on visit 2 (sputum V2).

Detailed methods for bronchoscopy, SBP-Ag, and BAL cell preparation have previously been described [25]. A graded inhaled Ag challenge with *Dermatophagoides farinae* (Der p 1, dust mite), *Felis catus domesticus* (Fel d 1, cat) or *Ambrosia artemisiifolia* (Amb a 1, ragweed), obtained from Greer Laboratories (Lenoir, NC, USA) was performed to determine the participant’s AgPD_20_ (the provocative dose of Ag leading to a 20% fall in FEV_1_). One month later, a baseline bronchoscopy with BAL (4 × 40 ml aliquots of sterile 0.9% NaCl) was performed followed by administration of Ag at a dose of 20% of the AgPD_20_ into the lavaged segment (BAL V1, Figure 1). Bronchoscopy with BAL was repeated 48 h later (BAL V2, Figure 1). One month after the first SBP-Ag, a single dose of anti-IL-5 (750 mg, mepolizumab generously donated by GlaxoSmithKline) was administered intravenously to reduce the number of airway EOS. One month after mepolizumab treatment, the second SBP-Ag was performed with BAL immediately before (BAL V3) and 48 h later (BAL V4). Circulating EOS number was <100 per µl at the time of the second SBP-Ag (BAL V3). BAL cells were assessed by hemocytometer using Turk’s counting solution containing acetic acid and methylene blue and cell differentials were determined on cytospin preparations stained with the Wright-Giemsa-based Hema-3 (ThermoFisher/Thermo Scientific, Rockford, IL, USA). On BAL V2 (Figure 1), airway EOS were purified from BAL cells using a two-step Percoll gradient as previously described [8]. EOS were collected from the 1.085/1.100 g/ml interface.

### Induced Sputum

Induced sputum was obtained as previously described [22] on visit 1 (sputum V1, Figure 1) and 48 h after a graded WLAC leading to a 20% fall in FEV_1_ (sputum V2). Briefly, patients inhaled nebulized 3% saline for 5 minutes, rinsed out their mouth and then coughed with collection of the sputum produced. This was repeated a total of three times. The sputum was diluted 1∶1 with PBS. After centrifugation, cytospins were prepared and stained with Giemsa to determine cell distributions. Cells were stored in Trizol solution for RNA purification.

### RNA Preparation and Microarray Hybridization

Total RNA was extracted from BAL cells or purified BAL EOS using the RNeasy Mini Kit (Qiagen, Valencia, CA, USA), treated with DNase (RNase-free DNase kit, Qiagen), and RNA from BAL cells were submitted to the University of Wisconsin-Madison Gene Expression Center (Madison, WI) for labeling and hybridization. RNA quality and integrity was evaluated via Agilent 2100 Bioanalyzer platform (Agilent Technologies). Sense-strand cDNA was generated from total RNA using the Ambion Whole Transcript (WT) Target Expression Kit (Life Technologies, Grand Island, NY, USA). Fragmentation and labeling of the single-stranded cDNA was performed using the Affymetrix GeneChip WT Terminal Labeling Kit (Affymetrix, Santa Clara, CA, USA). The labeled cDNAs were hybridized to Human Gene ST 1.0 GeneChips (Affymetrix) according to the manufacturer’s protocols.

Induced sputum samples were shipped to Miltenyi Biotech for RNA extraction using standard RNA Trizol extraction protocols. Sample integrity was evaluated via Agilent 2100 Bioanalyzer platform (Agilent Technologies). 50 ng of RNA was amplified and labeled using the Aglient Low Input Quick Amp Labeling Kit (AgilentTechnologies). Hybridization was performed using the Agilent Gene Expression Hybridization Kit to Agilent Whole Human Genome Oligo Microarrays 8X60K for microarray analyses. The Agilent Feature Extraction Software was used to read out and process the microarray image files.

### Microarray Data Analysis

For BAL cells, raw data (CEL files) were uploaded into ArrayStar software version 4.0 (DNASTAR Inc., Madison, WI, USA) for normalization and statistical analysis. The robust multichip analysis (RMA) algorithm was used for background correction, quantile normalization and median polish summarization. Microarrays are deposited in the Gene Expression Omnibus repository (accession number: GSE46159). Four pairs of data comparisons that might reveal genes up-regulated in BAL cells after allergen challenge with or without mepolizumab were performed. The transcripts were filtered on the basis of ≥2-fold difference. The 99 genes up-regulated by SBP-Ag and decreased 1.5-fold by mepolizumab (Table S5) were determined using both the direct fold change between BAL V4 and V2 and the fold change between BAL V2 and V1 divided by the fold change between BAL V4 and V3 (see Figure 1).

For induced sputum samples, raw data files were analyzed using the Agilent Feature Extraction Software (FES) (Agilent Technologies). The software was used to determine feature intensities, perform background subtraction, reject outliers, and calculate statistical confidences. Microarrays are deposited in the Gene Expression Omnibus repository (accession number: GSE46238).To determine differential gene expression, the data outputted from FES was then analyzed using the Rosetta Resolver gene expression data analysis system (Rosetta Biosoftware). Ratios were calculated as control vs. sample, or before and after allergen challenge, by division of sample intensity through control signal intensity.

### Real-time qPCR

The reverse transcription reaction was performed using the Superscript III system (Invitrogen/Life Technologies, Grand Island, NY, USA). mRNA expression was determined by qPCR using SYBR Green Master Mix (SABiosciences, Frederick, MD, USA) and human IL5RA, LTC4S, FFAR2, CNR2, ARAP3, DAPK2, PRSS33, TNFSF14, IL1R1 and FCER2 (CD23ß) forward and reverse specific primers (see Table S1 for primer sequences) were designed using Primer Express 3.0 (Applied Biosystems, Carlsbad, CA, USA) and blasted against the human genome to determine specificity using http://www.ncbi.nlm.nih.gov/tools/primer-blast. The reference gene, ß-glucuronidase ((GUSB), forward: caggacctgcgcacaagag, reverse: tcgcacagctggggtaag), was used to normalize the samples. Standard curves were performed and efficiencies were determined for each set of primers. Efficiencies ranged between 91 and 96%. Data are expressed as fold change using the comparative cycle threshold (ΔΔCT) method as described previously [9]. The values presented in Figure 4 are fold change = (2^−ΔΔCt^) compared to expression in BAL cells at V1 (before SBP-Ag).

### Statistical Analyses

To compare expression of genes in total BAL cells and purified BAL EOS by qPCR, data were log transformed and analyzed using the Student’s paired *t*-test. Statistical analyses were performed using SigmaPlot 11.0 software package.

## Results

### Gene Expression Analysis in BAL Cells 48 h after SBP-Ag by Microarrays

**Figure 2:**
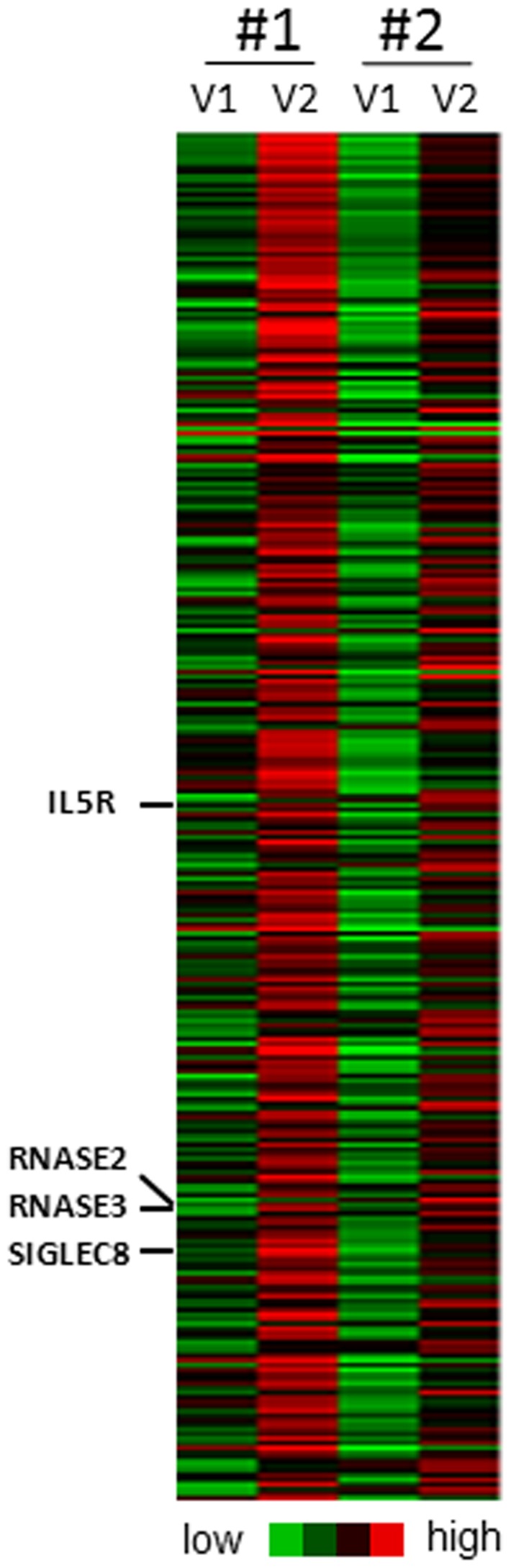
Heat map generated using Cluster 3.0 and presenting the 299 genes up-regulated by more than 2-fold in BAL cells from both subjects #1 and #2, 48 h after SBP-Ag (BAL V2 as shown in Figure 1) compared to before SBP-Ag (V1). Eosinophil-markers (IL5RA, RNASE2, RNASE3 and SIGLEC8) are highlighted. The full list of gene names is presented on Table S2.

While less than 1% of BAL cells were EOS before SBP-Ag, they increased to 73.9% ±4.2 (n = 8) of the total BAL cell population after SBP-Ag. After mepolizumab, SBP-Ag elicited a significant but much attenuated increase in EOS counts compared to before mepolizumab (34% ±9 versus 73.9% ±4.2; p<0.001, paired *t* test, n = 8) [26]. 2 of 8 subjects (Figure 2) were randomly chosen for gene expression analysis using a whole human genome transcript microarray. For these 2 subjects, EOS constituted 72 and 74% of the BAL cells after SBP-Ag and were reduced to 24 and 39% of the BAL when SBP-Ag was performed after mepolizumab.

In total BAL cells, expression of 299 genes was up-regulated by more than 2-fold 48 h after SBP-Ag (Figure 2 and Table S2). In both subjects, 22 transcripts were increased by more than 5-fold (Table S3) and 4 transcripts by more than 10-fold (ALOX15, CD1A, CD24 and FAM101B). The 299 genes up-regulated after SBP-Ag included marker genes for EOS (IL5RA, RNASE2, RNASE3, and SIGLEC8), and other genes well-known to be expressed by EOS (ADAM8, CCR3, CD69, CDA, GATA1, HRH4, ICAM3, IL1RL1, LTC4S, OXER1, P2RY2, PADI4, and SIGLEC10). Genes associated with lung remodeling were also found such as metalloproteinase genes (MMP12, MMP9), as well as some IL-13-induced chemokines (CCL13, CCL2) and Th2-associated genes (CCL22, FCER2, and IL1R2) [27]–[29]. While expression of genes coding for soluble mediators (e.g. cytokines) were unchanged, receptors for several mediators known to participate in lung fibrosis were up-regulated (CCR2, FGFR2, FLT1, IGF1R, IL1R1 and IL6R) [27], [29], [30]. 19 transcripts mostly expressed in dendritic cells, lymphocytes or mast cells (Table S4), Swissprot research at http://www.uniprot.org/) were also included in the 299 transcripts. Further analysis using DAVID Bioinformatics Resources indicated that 31 genes have been previously associated with asthma, allergy or a pulmonary disease (DAVID Bioinformatics Resources 6.7; National Institute of Allergy and Infectious Diseases (NIAID), NIH) (Table S5).

99 (33.1%) of the 299 transcripts elevated by more than 2-fold in BAL cells after SBP-Ag were decreased by more than 1.5-fold after reduction of EOS by mepolizumab (Table S6). Of note, 1.5- versus 2-fold reduction was chosen because mepolizumab decreased EOS percentage in BAL cells by ∼50%. Among the 99 genes whose expression was down-regulated after SBP-Ag are the well-known EOS-specific genes (IL5RA, RNASE2, RNASE3 and SIGLEC8) and all the other transcripts mentioned above as previously known to be expressed by EOS (ADAM8, CD69, GATA1, HRH4, ICAM3, IL1RL1, LTC4S, OXER1, P2RY2, PADI4 and SIGLEC10) except for CCR3. In addition, other genes that are not known to be expressed by EOS were down-regulated by mepolizumab. Whether these genes are expressed by other cell types (lymphocytes, dendritic cells, macrophages, or mast cells) that are affected directly or indirectly by the lack of IL-5 or the reduction of EOS or directly expressed by EOS is not known.

### Identification of EOS-associated Genes by Microarray Using Induced Sputum after WLAC

Sputum cells from 6 subjects were analyzed by a whole genome transcript microarray. WLAC increased the percentage of EOS in sputum from 2.0 to 8.2% (p<0.001). Marker genes for EOS (RNASE2, RNASE3, IL5RA and SIGLEC8) were increased by more than 2-fold after WLAC in 3 of the 6 subjects involved in this analysis. Sputum samples from these 3 individuals allowed us to establish a list of 365 EOS-associated genes whose expression was increased by more than 1.5-fold after WLAC (Table 1). In addition to the original EOS markers (RNASE2, RNASE3, IL5RA and SIGLEC8), the 365 transcripts (Table 1) included some other well-known genes expressed by EOS. In fact, 11 of the 13 genes (ADAM8, CCR3, CD69, CDA, HRH4, ICAM3, IL1RL1, LTC4S, P2RY2, PADI4 and SIGLEC10) known to be expressed by EOS and up-regulated in BAL cells after SBP-Ag (Table S2) were also seen in sputum after WLAC.

**Table 1:** EOS-associated genes in sputum after WLAC.

AATK	CD48	DNAJB5	GPR56	KCNK5	NKX6-2	RAB37	ST6GAL1
ABCB1	CD69	DNASE1L3	GPR84	KCNMB4	NOV	RAB3D	ST8SIA4
ABLIM2	CD93	DUSP2	GPR97	KCNN4	NPDC1	RASAL1	STAB1
ACAP1	CDA	DYSF	GPT	KCTD15	NRARP	RASSF2	SULF2
ACOX2	CDKN1C	ECE1	GRASP	KIF21B	NRG1	RASSF5	SYNE1
ACPP	CDRT8	EGR3	GRB10	KLF10	NTRK1	RD3	SYNE2
ADAM19	CECR6	EGR4	GSDMA	KRTAP19-1	ODF4	RGAG4	TAC4
ADAM28	CHI3L1	EIF2C2	HAS1	LBH	OLIG1	RGL4	TAGAP
ADAM8	CHST15	EMB	HBA2	LGALS12	OLIG2	RGS16	TAS2R50
ADAMTS10	CHST2	EMR4P	HBB	LGALS2	OSBPL3	RGS2	TBC1D3B
ADORA2A	CHSY1	ENHO	HBD	LIMK2	OSM	RHOH	TESC
AGER	CLC	ENPP1	HCG27	LPPR2	P2RY14	RNASE2	TET2
AGPAT9	CLEC10A	ENPP2	HDAC4	LRG1	P2RY2	RNASE3	TGM2
ALPL	CLEC4F	EPHB3	HDC	LTC4S	P2RY6	RNASE6	THBS4
AMPD2	CLEC4G	ETV3	HGF	LTF	PADI2	RNF19B	TIAM2
ANKH	CLEC5A	F13A1	HHIP	LY9	PADI4	RUFY4	TIMP1
APOBEC3B	CMTM2	FAM101B	HIC1	MARCKSL1	PAK1	RUNX2	TMEM154
ARAP3	CNR2	FAM198B	HIF1A	MAST4	PALLD	RYBP	TMEM156
AREG	COL18A1	FAM20A	HIP1R	MATK	PARM1	S100B	TMEM71
ASB2	COMP	FAM46B	HIST1H1D	MBOAT7	PDCD1	S1PR1	TNFRSF10C
ASGR2	CORO1A	FAM65B	HIST1H1E	MCTP2	PDE2A	SATB1	TNFSF14
ATHL1	CREB3L3	FAM83A	HIST1H2AC	MECOM	PGLYRP1	SCHIP1	TPCN1
ATL2	CREB5	FCAR	HIST1H2AE	MGAM	PHF19	SCNN1D	TREML2
ATP2A3	CSGALNACT2	FCER1A	HIST1H2BO	MGC24103	PHLDB1	SDS	TRERF1
ATP8B4	CST7	FCER2	HIST1H4A	MMP1	PIK3R5	SELL	TRPM6
B3GNT8	CTTNBP2	FCGR2B	HIST2H2BE	MMP10	PIK3R6	SEMA4B	TSPAN18
B4GALNT3	CX3CR1	FCN1	HSPA6	MMP12	PIP5K1B	SEMA7A	TTYH2
BIRC3	CXCR1	FFAR2	ICAM3	MMP25	PLAUR	SIGLEC10	VDR
CACNA1E	CXCR2	FFAR3	IFITM1	MMP7	PLD4	SIGLEC8	VEGFA
CACNA2D3	CXCR2P1	FGF11	IGFL1	MPZL3	PLEKHG2	SLC16A10	VENTX
CALCRL	CYP1B1	FHL3	IL10	MRC2	PLUNC	SLC24A3	VNN2
CAMK1	CYP4F12	FLJ11710	IL18R1	MRVI1	PPP1R14A	SLC26A8	VSTM1
CASS4	CYSLTR2	FNDC1	IL1R1	MSRB3	PPP2R2C	SLC2A13	WNT5A
CBFA2T3	CYTIP	FRY	IL1R2	MST4	PRDM1	SLC3A1	WTAP
CCL17	DAB2IP	FZD2	IL1RAP	MT1G	PRKCB	SLC40A1	XYLT1
CCL26	DACH1	GADD45A	IL1RL1	MTVR2	PRSS33	SLC6A10P	YPEL3
CCR3	DAPK2	GAGE7	IL21R	MXD1	PSTPIP1	SLC6A8	ZBTB46
CD177	DCHS1	GAS6	IL4	NCAM2	PTGIR	SLC7A5	ZBTB46
CD1A	DEFB122	GATA2	IL5RA	NCRNA00085	PTGS2	SNAI1	ZDHHC18
CD1B	DEFB130	GCOM1	IL6R	NCRNA00152	PTP4A3	SNHG3	ZDHHC8
CD1C	DENND3	GFRA2	INSIG1	NCRNA00287	PTPN7	SOCS2	ZNF395
CD1E	DGKA	GGT5	IRX6	NDE1	PVRL2	SORBS1	ZNF469
CD207	DGKD	GLYCTK	ITGB1BP2	NDEL1	PXN	SORL1	ZNF507
CD209	DIRAS1	GPR160	JHDM1D	NECAB2	PYROXD2	SPATA9	
CD226	DNAH17	GPR183	KCNH2	NHSL2	QPCT	SPNS3	
CD300LB	DNAJB1	GPR35	KCNJ15	NKD1	RAB33A	SPOCK1	

365 genes upregulated by more than 1.5 in each of the 3 subjects with high levels of EOS-characterizing genes (IL5RA, RNASE2, RNASE3 and SIGLEC8) after WLAC.

### Identification of Airway EOS Gene Expression

**Figure 3:**
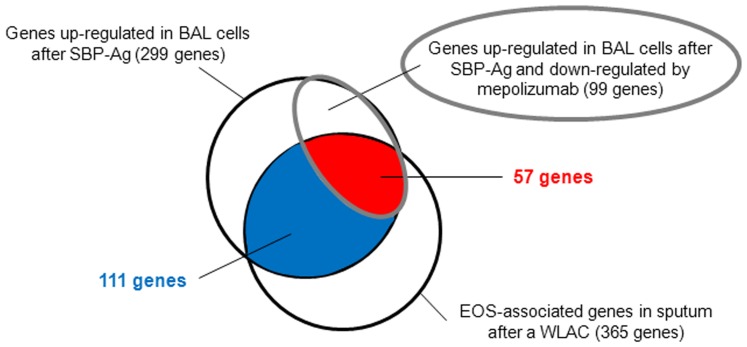
Venn diagram identifying 57 genes highly associated with airway EOS after an allergen challenge. 57 genes defined at the intersection of genes up-regulated in BAL cells after SPB-Ag, down-regulated when SBP-Ag was performed following mepolizumab, and part of the EOS-associated genes in the sputum following WLAC.

We have utilized 3 airway models that either augmented or reduced the EOS population, and defined genes concomitantly expressed with markers of EOS genes in the context of allergen challenges *in vivo*. Figure 3 shows that 168 (111+57) (46%) of the 365 EOS-associated genes in sputum were also identified in the 299 genes up-regulated in BAL cells. This suggests that despite distinct anatomical sites and provocative protocols, infiltrating EOS express a consistent group of transcripts. Furthermore, the intersection of these 2 sets with the genes decreased by mepolizumab identified a group of 57 genes (Figure 3 and Table 2). We propose that this intersection represents a stringent selection for gene expression by airway EOS. A literature search in PubMed (http://www.ncbi.nlm.nih.gov/pubmed/) indicates that only 16 of the 57 genes (28%) have been previously linked to EOS. Therefore, 41 genes (>70%) in this subset have not previously been associated with EOS.

**Table 2:** 57 genes upregulated in BAL cells after SBP-AG, down-regulated when SBP-Ag was performed following mepolizumab, and part of the EOS-associated genes in sputum after WLAC.

ADAM28	**CNR2** *	FFAR3	LGALS12	**PRSS33** *	SPNS3
ADAM8	CORO1A	FHL3	**LTC4S** *	RAB37	TESC
**ARAP3** *	CYP4F12	GGT5	MCTP2	RAB3D	**TNFSF14** *
ASB2	DACH1	GPR56	MMP25	RD3	TREML2
ATP2A3	**DAPK2** *	GPR97	NHSL2	RNASE2	TRPM6
CASS4	DGKD	ICAM3	P2RY2	RNASE3	TSPAN18
CD300LB	EMR4P	IFITM1	PADI2	SIGLEC10	VSTM1
CD69	FAM101B	IL1RL1	PADI4	SIGLEC8	
CDA	FAM65B	**IL5RA** *	PGLYRP1	SLC24A3	
CHST15	**FFAR2** *	KIF21B	PIK3R6	SORL1	

* genes chosen for validation of the list by qPCR.

IL5RA, RNASE2, RNASE3 and SIGLEC8 are known markers of EOS.

### Validation of the Airway EOS-associated Genes

**Figure 4:**
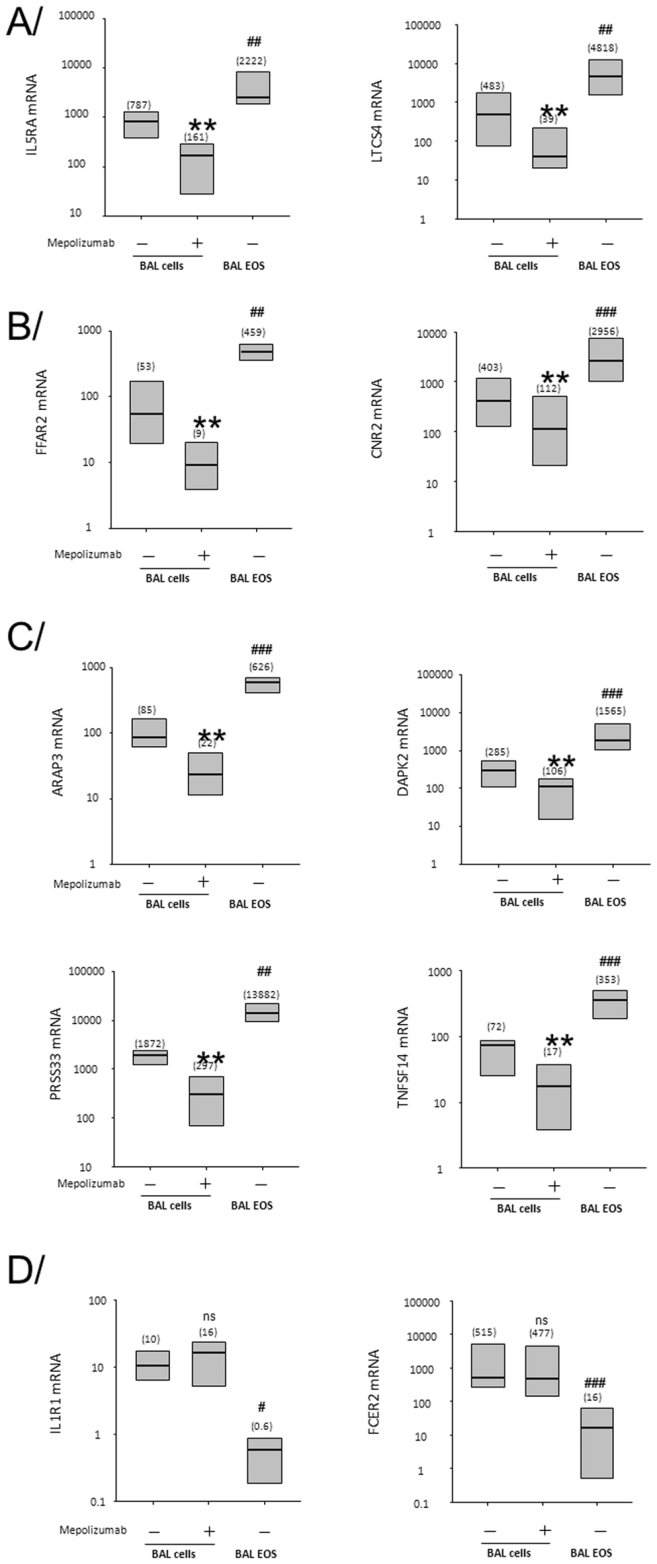
Validation by qPCR of the 57 genes associated with airway EOS after airway allergen challenge. qPCR was performed on BAL cells before SBP-Ag (BAL V1 as shown on Figure 1), 48 h after allergen with (V4) or without mepolizumab treatment (V2), and on purified BAL EOS (BALEOS) after allergen challenge (no mepolizumab, V2). Fold changes compared to expression in BAL cells before allergen challenge and mepolizumab (V1) are presented. Box plots depict the median and the interquartile range between the 25^th^ and the 75^th^ percentiles. 6 subjects are included in each group and means are presented in parentheses. ** indicates that mRNA level in BAL cells is significantly decreased by mepolizumab compared to BAL cells on V2 (p<0.01). # indicates that mRNA level in BAL EOS at V2 is significantly higher or lower compared to total BAL cells on V2 (^#^, p<0.05; ^##^, P<0.01: ^###^, P<0.001).

To validate the expression of these genes by airway EOS, we performed qPCR for 8 of the 57 genes (Table 2). The samples from the 6 subjects not used in the microarray analyses were analyzed by qPCR. Gene expression in highly purified BAL EOS (BAL V2, see Figure 1) and total BAL cells after SBP-Ag before (BAL V2) and after mepolizumab infusion (BAL V4) are presented as fold change (2^−ΔΔCt^ method) compared to gene expression in BAL cells before SBP-Ag (BAL V1). Initially, 2 established genes expressed by EOS were chosen as controls (IL5RA and LTCS4). As shown in Figure 4A, expression of both genes was highly up-regulated by SBP-Ag, and strongly decreased (>80%) by mepolizumab. Both genes displayed very high levels in pure BAL EOS compared to total BAL cells, suggesting that EOS are likely the main source of IL5RA and LTCS4 in BAL cells obtained after SBP-Ag. In Figure 4B, FFAR2 and CNR2 were selected as genes that have been connected with asthma and/or allergy and have been reported to be expressed by EOS [31], [32]. Both genes exhibited the same profile as IL5RA and LTCS4, indicating that EOS are also an important source of FFAR2 and CNR2 in BAL cells. In addition, 4 of the 41 genes for which expression in EOS has not previously been described were randomly selected. As shown in Figure 4C, all 4 genes (ARAP3, DAPK2, PRSS33 and TNFSF14) presented the same profile of expression as IL5RA, LTCS4, FFAR2 and CNR2 demonstrating that these transcripts are highly expressed in airway EOS. IL1R1 and FCER2 (CD23ß) were chosen as negative controls as they were up-regulated in unseparated BAL cells after SBP-Ag (Table S2) and part of the EOS-associated genes as established in sputum (Table 2), but were not affected by mepolizumab (Table S6). Conversely to IL5RA, LTCS4, FFAR2, CNR2, ARAP3, DAPK2, PRSS33 and TNFSF14, IL1R1 and FCER2 displayed very low expression in BAL EOS compared to total BAL cells (Figure 4D). Figure 4 validates the data obtained by microarrays and demonstrates that BAL EOS are likely the source of the 57 genes that increased post SBP-Ag in BAL cells. Using DAVID Bioinformatics Resources, genes were clustered according to their known functions. Six genes have been associated with a respiratory disease, 7 genes produce secreted proteins and 27 genes code for plasma membrane protein. Some other important clusters included cell adhesion (7 genes), immune and defense response (6 genes), and receptors for a variety of stimuli such as carbohydrates and nucleotides (15 genes) (Table S7).

## Discussion

We have used a unique approach combining 3 whole genome expression microarrays (sputum cells after WLAC, BAL cells after SBP-Ag, and BAL cells after mepolizumab and SBP-Ag) to identify 57 genes highly expressed by airway EOS after an allergen challenge. We demonstrated that EOS are likely the main source of these 57 genes expressed by total BAL cells. The 57 genes included genes such as CD69, IL5RA, LTC4S, RNASE2, RNASE3, SIGLEC8, ADAM8, CDA, ICAM3, IL1RL1 (ST2), and P2RY2 that are known to be expressed in EOS, and other genes not previously linked to EOS. Cluster analysis indicated these genes might have an impact on EOS migration and activation, as well as the innate and adaptive immune responses.

Of the 57 transcripts associated with airway EOS, 16 have been previously detected in circulating EOS, including IL1RL1, CNR2, and FFAR2. IL1RL1 (also known as ST2) is the receptor for IL-33, a cytokine that is released following epithelial damage during an allergic reaction. IL-33 can activate CD4^+^ T cells, type-2 innate lymphoid cells, and mast cells, as well as EOS [33]. The function of IL-33 on circulating EOS includes induction of degranulation, cytokine release, and increased survival. *In vivo*, blockade of the IL-33/IL1RL1 pathway reduces allergic inflammation and airway responsiveness [34]. Our study suggests that EOS might be the main source of IL-33 receptor among the airway inflammatory cells present after SBP-Ag, and EOS could contribute to IL-33-mediated effects in allergic asthma. CNR2 (also called CB2 receptor) is the cannabinoid receptor expressed in lymphoid organs [35]. CNR2 is known to be expressed by circulating EOS, and an endogenous ligand (2-arachidonoylglycerol) induces EOS migration [36]. In mice, genetic deletion of Cnr2 exacerbated contact allergic inflammation and influenza-induced excessive airway injury possibly via induction of pro-inflammatory and pro-remodeling mediators (IL-17, IL-13, TNF-α, GM-CSF) [37], [38]. CNR2 agonists reduced bronchoconstriction, mast cell degranulation and pulmonary inflammation in guinea pigs [39]. Another endogenous CNR2 ligand, anandamide is increased in BAL fluids after SBP-Ag, and correlated with the number of EOS in the airway [40]. Finally, FFAR2 expression by EOS has only been reported once [32]. FFAR2 (also known as GPR43) is a lipid G-protein coupled receptor for short-chain fatty acids that is produced by bacteria after fermentation of dietary fibers. Interestingly, the lack of FFAR2 led to unresolved inflammation in several animal models of inflammation, including allergen-induced airway inflammation [32], suggesting FFAR2 activation by short-chain fatty acids would be beneficial to reduce asthma.

One example of potentially novel EOS-derived mediators is TNFSF14 (also called LIGHT), a membrane-expressed or secreted protein that interacts with TNFRSF14 membrane receptor (HVEM), and was characterized as a co-stimulatory ligand for lymphoid cells [41]. As most of the co-stimulatory members of the TNF/TNFR superfamily, TNFSF14 has been studied for its impact on autoimmune disease and transplant rejection [41]. More recently, LIGHT has been implicated in induction of airway fibrosis and smooth muscle hyperplasia via TGF-ß and IL-13 production in an allergic animal model [42]. Interestingly, in human sputum, LIGHT protein level has been associated with lower lung function (FEV_1%_ predicted) [43].

Another example of novel mediators in human EOS is PGLYRP1 or PGRP-S (also called Tag7), which recognizes bacterial peptidoglycan and has an antibacterial function [44]. PGLYRP1 is a 21Kd protein with a signal peptide for secretion. While PGLYRP1 has not been described in human EOS, bovine and mouse EOS are known to express and store the bovine and murine ortholog of PGLYRP1 [45], [46]. In addition, to its antimicrobial function, several mouse models using PGLYRP1 knock-out, have demonstrated both an anti-inflammatory role for PGLYRP1 that down-regulated the production of type-1 cytokines and chemokines [47], and a pro-inflammatory role in atopic dermatitis, contact dermatitis, and pulmonary inflammation models promoting the Th1, Th2 and Th17 response [48], [49]. Also, through its interaction with Hsp70 on cytotoxic lymphocytes, PGLYRP1 possesses anti-tumor cell activity [50].

Another intriguing gene is PRSS33 (also called Protease EOS). This member of the trypsin-like serine proteases has been described to be predominantly produced by macrophages [51]. However, an international patent has described PRSS33 as expressed by EOS as well (Pub. No.: WO/2001/016290). Proteases have crucial roles in maintaining homeostasis and regulating inflammation and immune reactions, thus their expression in EOS is under active investigation. For instance, PRSS21 (esp-1) has been found in EOS but not in neutrophils [52], and we have shown very recently that EOS could generate high amounts of another protease, MMP-9 [53]. Yet, the production of PRSS33 by human EOS remains uncertain.

There is little overlap between airway EOS genes described in the present study and previous microarrays using *in vitro* activated blood EOS. *In vitro*, IL-5 induced the up-regulation of CD69 by blood EOS [13], [14]. Surprisingly, CD69 was the only common gene between our 57 EOS-associated genes and the genes up-regulated by IL-5 or GM-CSF in these 2 previous studies. This suggests that a short-term *in vitro* activation using IL-5 or GM-CSF might not be an adequate model to reflect *in vivo* EOS activation after an allergen challenge. In addition, the present study analyzed the presence of genes associated with eosinophilia while the 2 previous studies analyzed genes that are cytokine-induced. Thus, in addition to their exposure to cytokines, airway EOS likely become activated as a result of their transmigration out of the blood and through the tissues. Future studies will be required to determine if the 57 genes are inducible or constitutively expressed by airway EOS. Comparison of gene expression in airway EOS after allergen challenge with blood EOS obtained from individuals who do not receive challenge would provide information on the inducible versus constitutive expression of these genes.

In a study by Nakajima et al. [54], microarray analysis examined EOS-specific transcripts by comparison to other nucleated peripheral blood cells. Among the 30 EOS-specific genes, 3 were present in our list of 57 genes (RNASE2, IL5RA and CNR2). 11 of the 30 genes were also up-regulated in our BAL cells after SBP-Ag including GPR44, ALOX15, P2RY14, IDO1, ADORA3, CAMK1, CCR3 and P2RY10; while 10 of the 30 genes were part of the EOS-associated genes in induced sputum (CLC, PLAUR, MARCKSL1, P2RY14, CAMK1, CCR3, and OLIG2).

Gene expression analysis by microarrays has been previously performed on bronchial biopsies from asthmatic patients and on airway epithelial cells [55], [56] but microarray analysis using BAL cells after *in vivo* challenge has not been reported. In our whole genome expression analysis, 299 genes were up-regulated after SBP-Ag in BAL cells. Cluster analysis by DAVID Bioinformatics Resources identified 36 genes involved in defense, immune, or wound-healing responses, 32 genes implicated in cell adhesion or locomotion, 23 genes that regulate transcription, 21 genes associated with apoptosis and 12 genes that are part of the second-messenger-mediated signaling. Importantly, the EOS markers (RNASE2 and RNASE3, also called EDN and ECP) as well as 15 other well-known EOS-associated genes were up-regulated concomitantly with EOS accumulation in the airway following SBP-Ag. Of these 17 genes, all but CCR3 were decreased by the mepolizumab infusion. Among the 13 genes up-regulated by SBP-Ag and related to lung remodeling (CCL2, CCL13, CCL22, CCR2, FCER2, FGFR2, FLT1, IGF1R, IL1R1, IL1R2, IL6R, MMP9 and MMP12), 11 of them were unchanged following mepolizumab. This suggests that in this model mepolizumab did not impact expression of remodeling-associated genes.

Potential limitations of this study include the inability to assess gene expression in non-hematopoietic airway cells (including epithelial cells) or to detect by microarray transcripts, such as cytokines, that are in low abundance. Unlike microarray analysis, qPCR allowed detection of IL-13 and IL-17A in BAL after SBP-Ag [9]. This suggests that some genes might have been excluded from the final eosinophilic genes and that the list of 57 genes is not an exhaustive inventory. Finally, it is important to note that a portion of the genes that are not affected by mepolizumab may in fact represent genes associated with a distinct phenotype of eosinophils that were recruited to or differentiated in the airway following administration of mepolizumab.

In conclusion, we have identified 57 genes highly expressed *in vivo* by airway EOS (BAL and sputum). Most of the 57 genes have not been previously studied in EOS and are important candidates to understand tissue EOS biology and to find new targets for therapies associated with an excessive eosinophilic response.

## Supporting Information

Table S1 **Primer sequences used for real-time PCR.**
(DOCX)

Table S2 **299 Genes upregulated in BAL cells 48 h after segmental allergen challenge.**
(DOCX)

Table S3 **22 Genes upregulated by more than 5 fold in both subjects in BAL cells 48 h after segmental allergen challenge.**
(DOCX)

Table S4 **Main cellular sources (non-eosinophilic) of the transcripts up-regulated in BAL cells after SBP-Ag.**
(DOCX)

Table S5 **Among the 299 genes upregulated in BAL cells after SBP-Ag, 31 have been previously genetically associated with asthma, allergy or a pulmonary disease.** DAVID Bioinformatics Resources 6.7; National Institute of Allergy and Infectious Diseases (NIAID), NIH; http://david.abcc.ncifcrf.gov/.
(DOCX)

Table S6 **99 genes up-regulated in BAL cells by SBP-Ag and down-regulated after mepolizumab.**
(DOCX)

Table S7 **Genes up-regulated in BAL cells after allergen challenge, down-regulated by mepolizumab and part of the EOS-associated genes in the sputum: functional annotation clustering (DAVID Bioinformatics Resources 6.7, National Institute of Allergy and Infectious Diseases, NIH).**
(DOCX)
