# Supplementary material for: Identification of Genes Expressed by Human Airway Eosinophils after an In Vivo Allergen Challenge
Source: PLoS One. 2013 Jul 2;8(7):e67560. doi: 10.1371/journal.pone.0067560 (PMC3699655; doi:10.1371/journal.pone.0067560)
Supplement: Table S1 — Primer sequences used for real-time PCR. (DOCX) [file pone.0067560.s001.docx]

**Table S1: Primer sequences used for real-time PCR**

| **GENE** | **Forward primer** | **Reverse primer** |
| --- | --- | --- |
| IL5R | aagtgtgcggaccatcctgca | ccaggagaccctggtggggc |
| LTC4S | gcccaggtgaactgcagcga | gccgccgccccttcatgaaa |
| FFAR2 | ggctgcgtcgaacttccgct | gccaaaactcgtgagggcgc |
| CNR2 | cccacaacacaacccaaagccttct | tggggtgggcccttcagattcc |
| IL1R1 | tgtgagcccagctaatgagaca | ccggtgacattacagatcaattgt |
| ARAP3 | gcccccgacagaagagagagca | tagccagcatcctcccgtgcc |
| DAPK2 | agagacccggaaacggctcaca | ccacagactccctgcgcacc |
| PRSS33 | aggtcctgctccttctggtgctg | tccgactggacatgcggggct |
| TNFSF14 | ctaggagagatggtcacccgcctg | ggttgacctcgtgagaccttcgc |
| CD23b | tggaacaagcagaatttagca | ctgggaagctcctcgatctc |
