# Supplementary material for: Identification of Genes Expressed by Human Airway Eosinophils after an In Vivo Allergen Challenge
Source: PLoS One. 2013 Jul 2;8(7):e67560. doi: 10.1371/journal.pone.0067560 (PMC3699655; doi:10.1371/journal.pone.0067560)
Supplement: Table S2 — 299 Genes upregulated in BAL cells 48 h after segmental allergen challenge. (DOCX) [file pone.0067560.s002.docx]

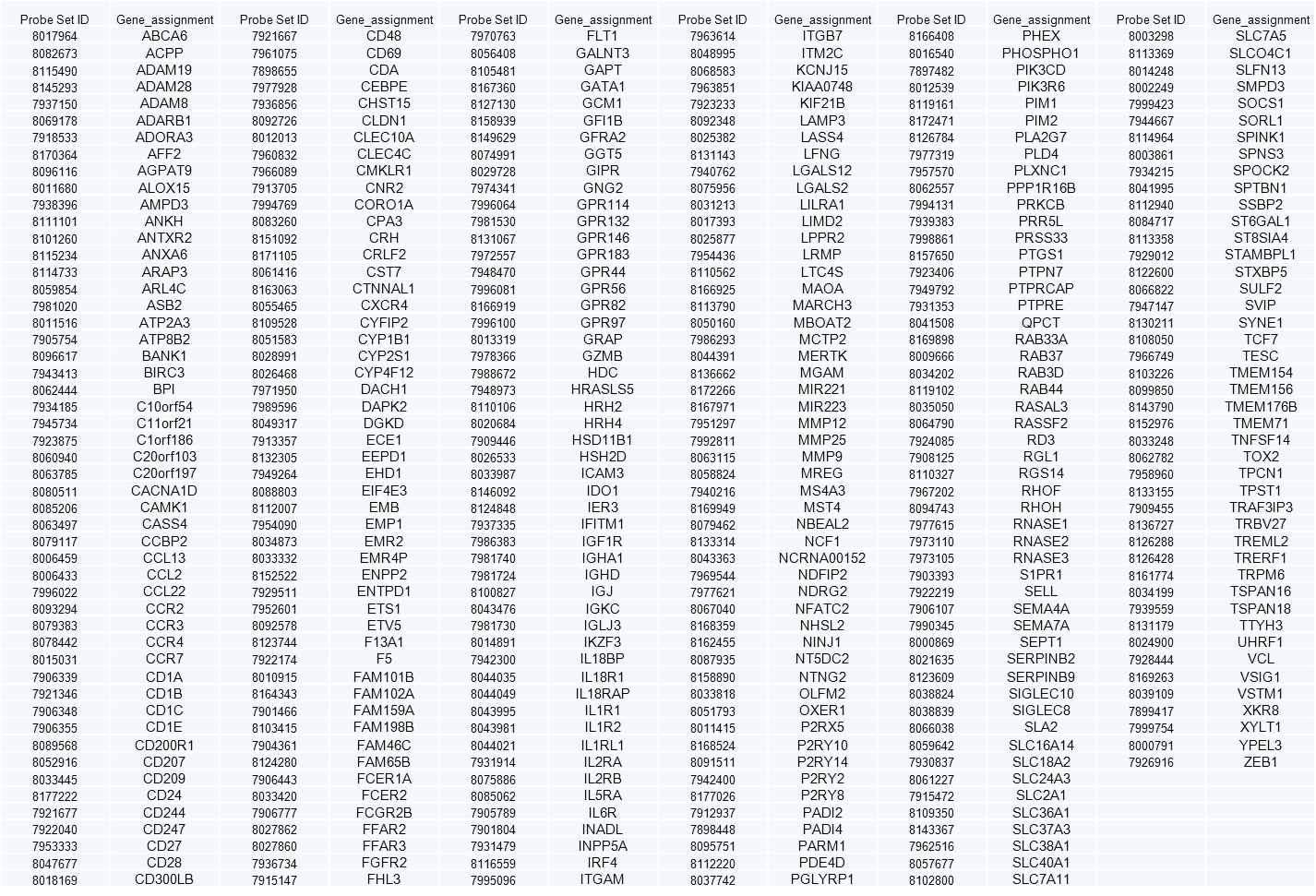


Eosinophil markers

**Table S2. 299 Genes upregulated in BAL cells 48 h after segmental allergen challenge**
