# Supplementary material for: Identification of Genes Expressed by Human Airway Eosinophils after an In Vivo Allergen Challenge
Source: PLoS One. 2013 Jul 2;8(7):e67560. doi: 10.1371/journal.pone.0067560 (PMC3699655; doi:10.1371/journal.pone.0067560)
Supplement: Table S3 — 22 Genes upregulated by more than 5 fold in both subjects in BAL cells 48 h after segmental allergen challenge. (DOCX) [file pone.0067560.s003.docx]

**Table S3. 22 Genes upregulated by more than 5 fold in both subjects in BAL cells 48 h after segmental allergen challenge**

| Probe Set ID | gene_assignment | Fold change, subject 1 | Fold Change, subject 2 |
| --- | --- | --- | --- |
| 7906339 | CD1A | 31.5 | 10.0 |
| 8006433 | CCL2 | 31.1 | 9.5 |
| 8082673 | ACPP | 20.9 | 9.8 |
| 8011680 | ALOX15 | 15.5 | 15.2 |
| 7912937 | PADI2 | 17.0 | 8.5 |
| 8010915 | FAM101B | 14.0 | 11.2 |
| 8177222 | CD24 | 10.7 | 12.1 |
| 8027860 | FFAR3 | 15.6 | 5.9 |
| 7918533 | ADORA3 | 15.1 | 6.3 |
| 8038839 | SIGLEC8 | 15.2 | 5.8 |
| 8011516 | ATP2A3 | 12.6 | 7.5 |
| 7906355 | CD1E | 5.3 | 13.3 |
| 7921346 | CD1B | 7.2 | 11.1 |
| 7996022 | CCL22 | 11.9 | 6.4 |
| 7937150 | ADAM8 | 11.3 | 6.9 |
| 8006459 | CCL13 | 10.5 | 7.7 |
| 8085206 | CAMK1 | 11.2 | 5.4 |
| 8033987 | ICAM3 | 8.0 | 6.5 |
| 7921677 | CD244 | 8.9 | 5.2 |
| 8012539 | PIK3R6 | 5.5 | 7.1 |
| 7898448 | PADI4 | 5.7 | 5.7 |
| 8027862 | FFAR2 | 5.2 | 6.0 |
