# Supplementary material for: Identification of Genes Expressed by Human Airway Eosinophils after an In Vivo Allergen Challenge
Source: PLoS One. 2013 Jul 2;8(7):e67560. doi: 10.1371/journal.pone.0067560 (PMC3699655; doi:10.1371/journal.pone.0067560)
Supplement: Table S4 — Main cellular sources (non-eosinophilic) of the transcripts up-regulated in BAL cells after SBP-Ag. (DOCX) [file pone.0067560.s004.docx]

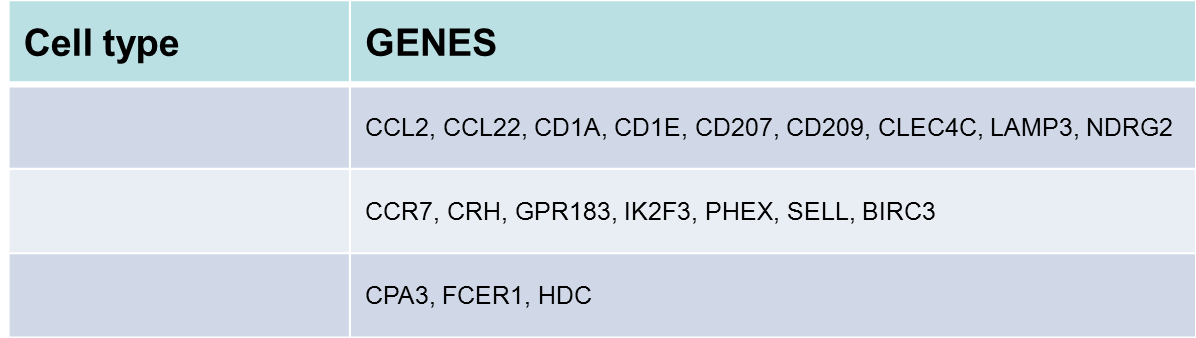


Dendritic cells

Lymphocytes

Mast cells

**Table S4. Main cellular sources (non-eosinophilic) of the transcripts up-regulated in BAL cells after SBP-Ag**

Swissprot research at http://www.uniprot.org/
