# Supplementary material for: Identification of Genes Expressed by Human Airway Eosinophils after an In Vivo Allergen Challenge
Source: PLoS One. 2013 Jul 2;8(7):e67560. doi: 10.1371/journal.pone.0067560 (PMC3699655; doi:10.1371/journal.pone.0067560)
Supplement: Table S5 — Among the 299 genes upregulated in BAL cells after SBP-Ag, 31 have been previously genetically associated with asthma, allergy or a pulmonary disease. DAVID Bioinformatics Resources 6.7; National Institute of Allergy and Infectious Diseases (NIAID), NIH; http://david.abcc.ncifcrf.gov/. (DOCX) [file pone.0067560.s005.docx]

**Table S5**. Among the 299 genes upregulated in BAL cells after SBP-Ag, 31 have been previously genetically associated with asthma, allergy or a pulmonary disease


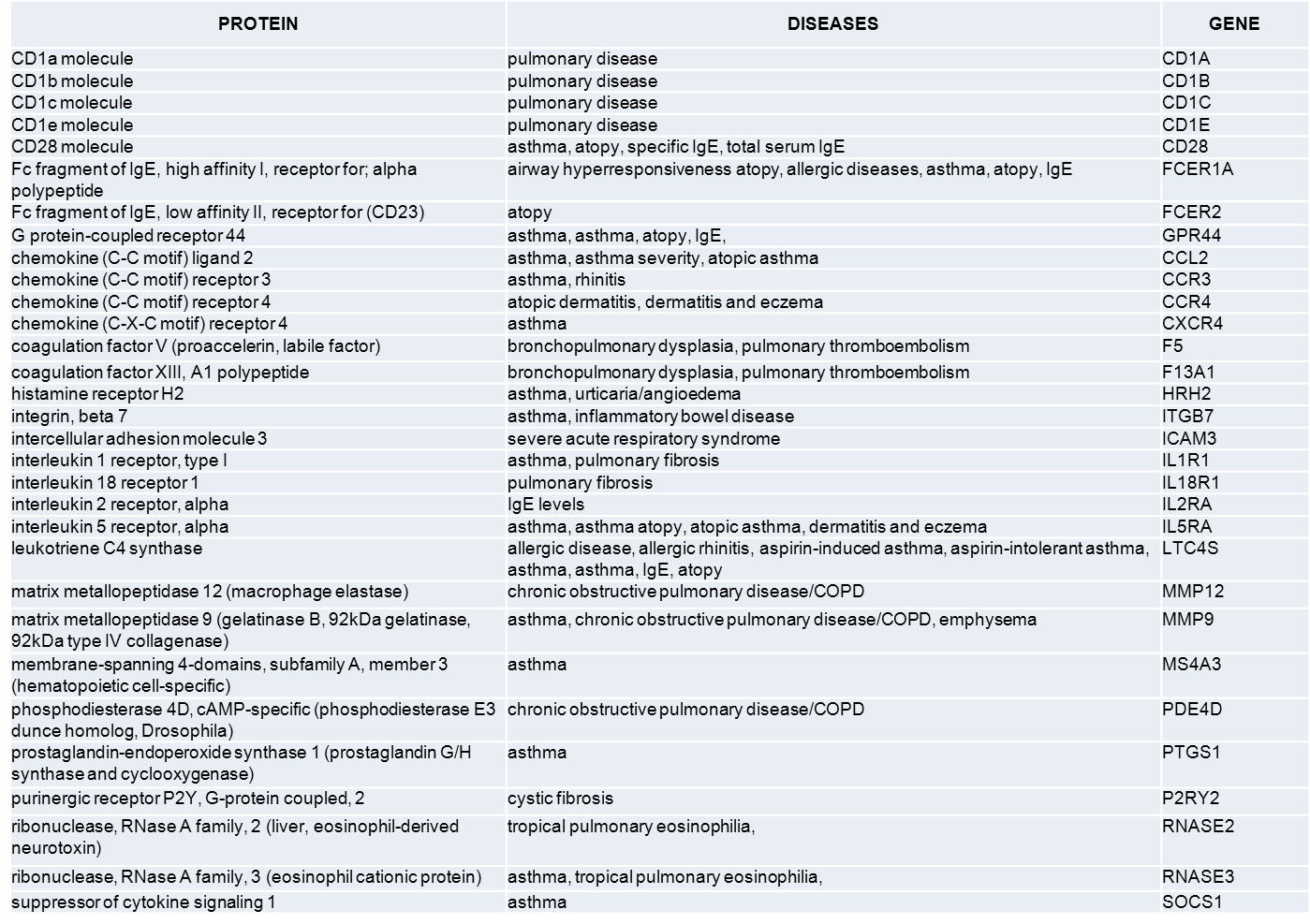


DAVID Bioinformatics Resources 6.7; National Institute of Allergy and Infectious Diseases (NIAID), NIH ; <http://david.abcc.ncifcrf.gov/>
