# Supplementary material for: Identification of Genes Expressed by Human Airway Eosinophils after an In Vivo Allergen Challenge
Source: PLoS One. 2013 Jul 2;8(7):e67560. doi: 10.1371/journal.pone.0067560 (PMC3699655; doi:10.1371/journal.pone.0067560)
Supplement: Table S6 — 99 genes up-regulated in BAL cells by SBP-Ag and down-regulated after mepolizumab. (DOCX) [file pone.0067560.s006.docx]

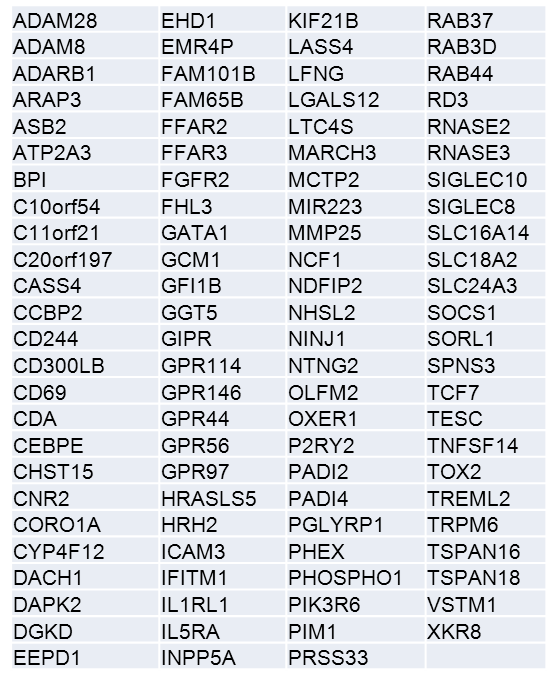


**Table S6.**

**99 genes up-regulated in BAL cells by SBP-Ag and down-regulated after mepolizumab**

EOS markers
