# Supplementary material for: Identification of Genes Expressed by Human Airway Eosinophils after an In Vivo Allergen Challenge
Source: PLoS One. 2013 Jul 2;8(7):e67560. doi: 10.1371/journal.pone.0067560 (PMC3699655; doi:10.1371/journal.pone.0067560)
Supplement: Table S7 — Genes up-regulated in BAL cells after allergen challenge, down-regulated by mepolizumab and part of the EOS-associated genes in the sputum: functional annotation clustering (DAVID Bioinformatics Resources 6.7, National Institute of Allergy and Infectious Diseases, NIH). (DOCX) [file pone.0067560.s007.docx]

**Table S7**.

**Genes up-regulated in BAL cells after allergen challenge, down-regulated by mepolizumab and part of the EOS-associated genes in the sputum: functional annotation clustering (DAVID Bioinformatics Resources 6.7, National Institute of Allergy and Infectious Diseases, NIH)**

**
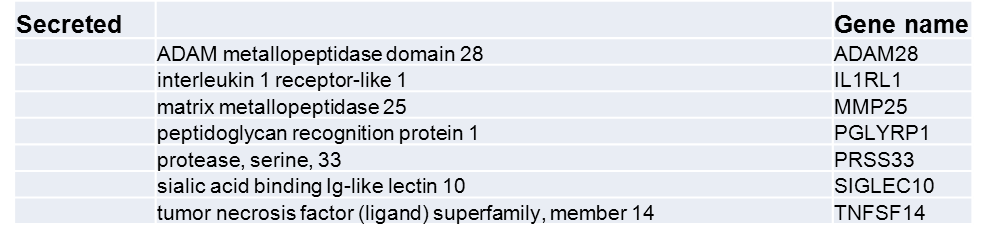

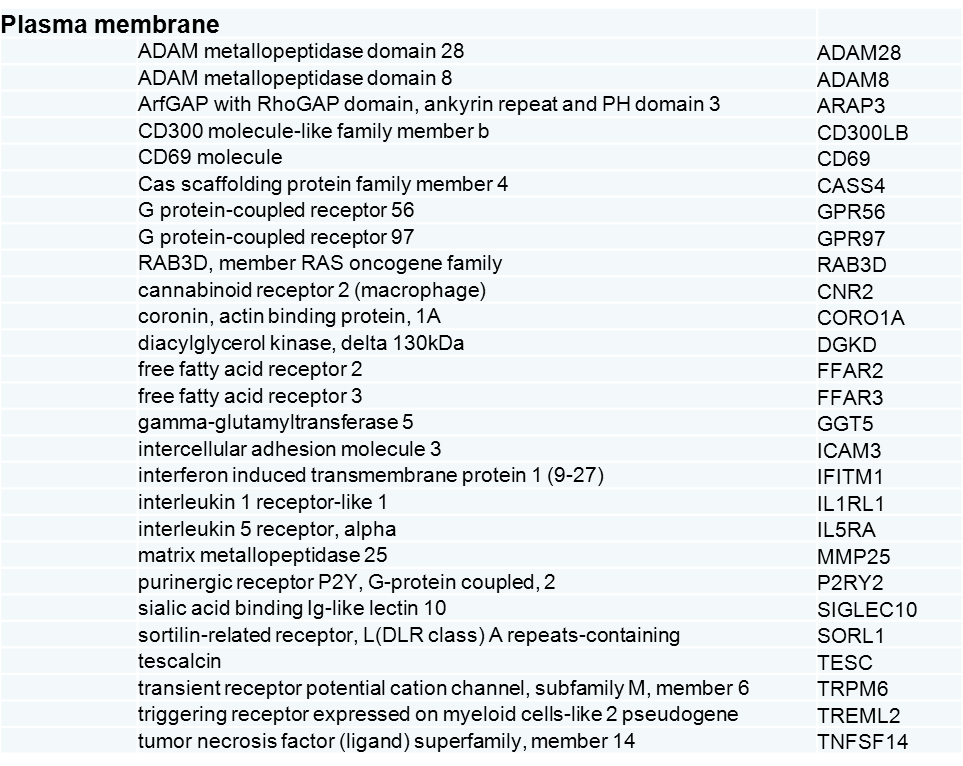
**

**Table S7**…

**… Table S7**


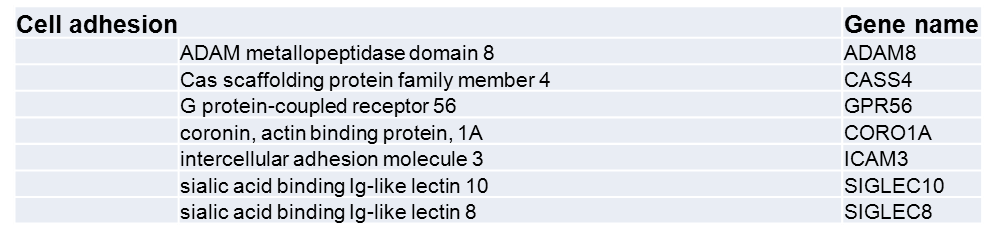

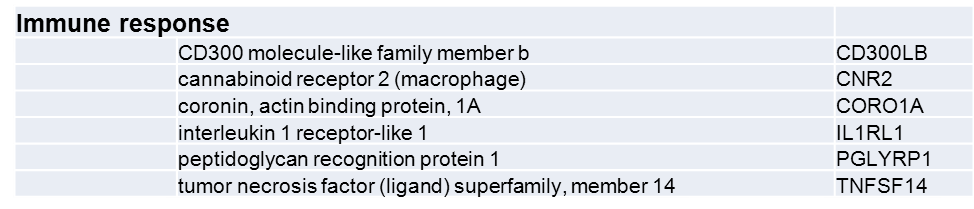

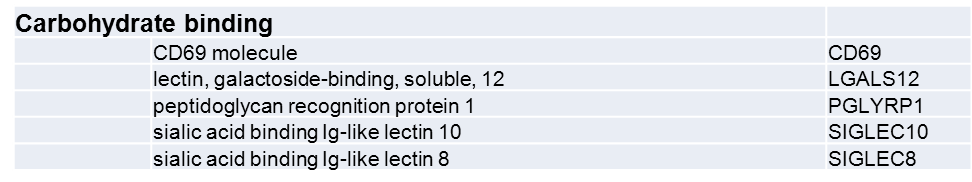

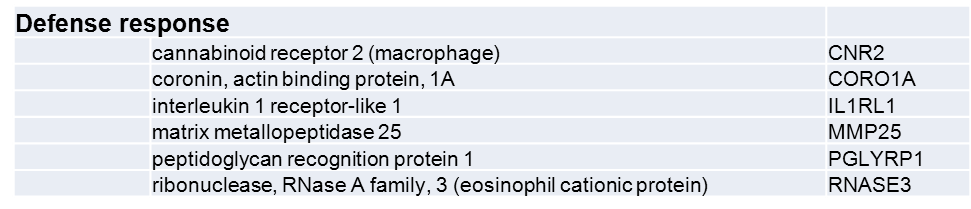


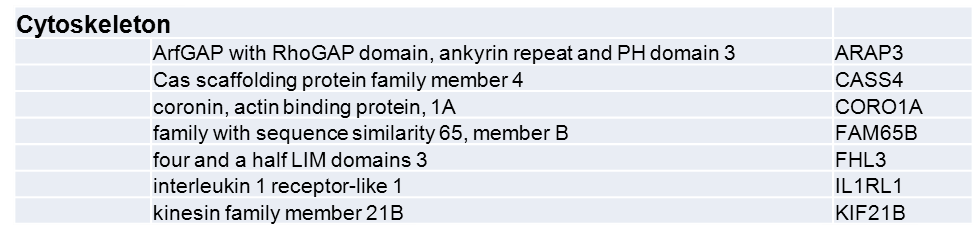
**
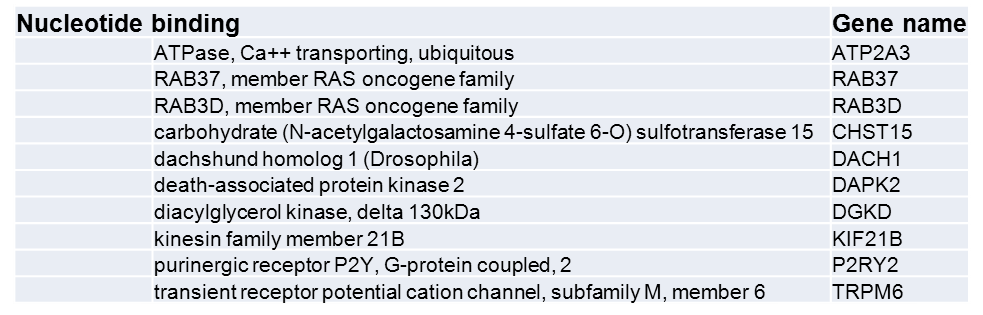
**


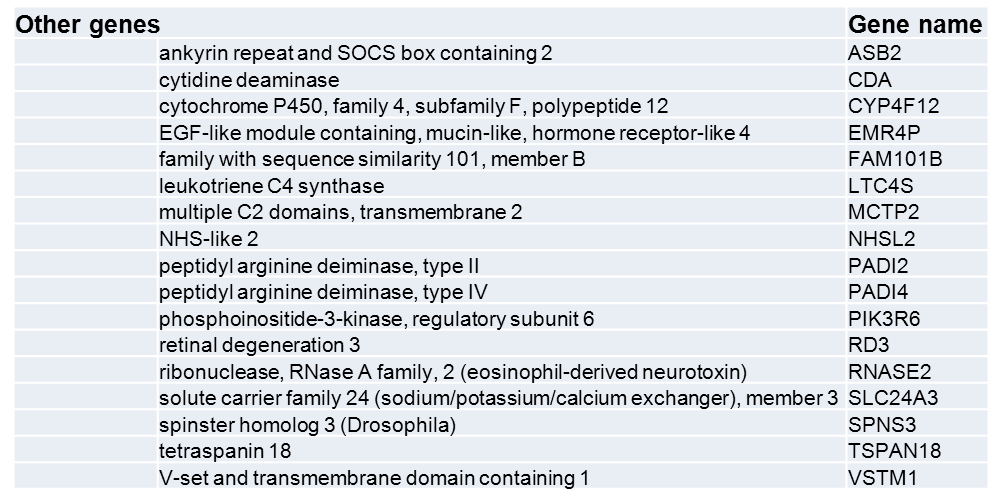

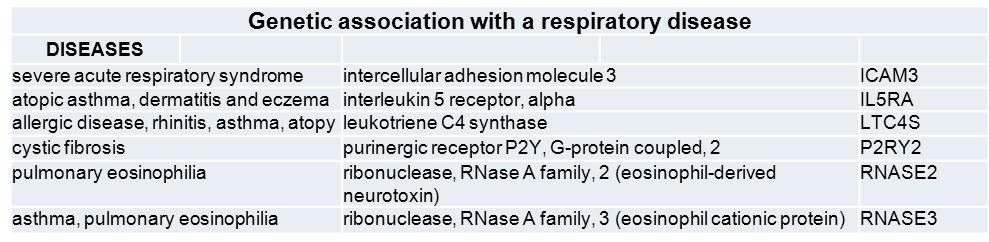


**…Table S7**
